# Association between TGFβ1 Levels in Cord Blood and Weight Progress in the First Year of Life

**DOI:** 10.3390/biomedicines11082220

**Published:** 2023-08-08

**Authors:** Noura Kabbani, Holger Stepan, Matthias Blüher, Thomas Ebert, Ronny Baber, Mandy Vogel, Wieland Kiess, Michael Stumvoll, Jana Breitfeld, Ulrike Lössner, Anke Tönjes, Susanne Schrey-Petersen

**Affiliations:** 1 Department of Obstetrics, University of Leipzig Medical Center, 04103 Leipzig, Germany; 2 Medical Department III—Endocrinology, Nephrology, Rheumatology, University of Leipzig Medical Center, 04103 Leipzig, Germany; 3 Helmholtz Institute for Metabolic, Obesity and Vascular Research (HI-MAG) of the Helmholtz Zentrum München, The University of Leipzig and University Hospital Leipzig, 04103 Leipzig, Germany; 4 Institute of Laboratory Medicine, Clinical Chemistry and Molecular Diagnostics, University of Leipzig Medical Center, 04103 Leipzig, Germany; 5 Leipzig Medical Biobank, University of Leipzig, 04103 Leipzig, Germany; 6 LIFE Child, Hospital for Children and Adolescents, Department of Pediatrics, University of Leipzig Medical Center, 04103 Leipzig, Germany; 7 Center for Pediatric Research (CPL), Hospital for Children and Adolescents, University of Leipzig Medical Center, 04103 Leipzig, Germany

**Keywords:** adipokine, TFGβ1, child growth, intrauterine growth, gestation

## Abstract

Transforming growth factor beta-1 (TGFβ1) is an adipokine secreted from adipose tissue, placental tissue and immune cells with a role in cell proliferation, cell apoptosis and angiogenic proliferation. The role of TGFβ1 in pregnancy and child growth and the source of cord TGFβ1 are yet unknown. In this study, we sought to clarify the correlation of TGFβ1 levels with parameters of intrauterine growth and child growth during the first year of life, and to determine whether their source is primarily of fetal or maternal origin. Serum samples and anthropometric measurements were obtained from the LIFE Child cohort of 79 healthy mother–child pairs. Measurements were conducted using enzyme-linked immunosorbent assays. Statistical analyses including Mann–Whitney U-test, correlation analyses and linear regression analyses were performed using GraphPad Prism and R. TGFβ1 levels were significantly higher in cord than in maternal serum, suggesting a fetal origin. Multivariate regression analyses revealed strong positive associations between cord TGFβ1 levels at birth and child weight at U6. Furthermore, cord TGFβ1 was significantly correlated with child weight at approximately one year of age. An increase of 10,000 pg/mL in cord TGFβ1 concentrations at birth was associated with a higher body weight of 201 g at roughly one year of age when adjusted for sex.

## 1. Introduction

Since the discovery of leptin, adipokines and their roles in various metabolic, endocrinological, immunogenic and vascular processes have been the topic of much research. Hundreds of adipokines have been identified and functionally characterized in recent years [1,2]. Especially of interest, is the function of adipokines in metabolic processes throughout pregnancy and the fetal period, as well as their implications in child growth [3]. The effects of intrauterine conditions on fetal development and cardiovascular risk were first examined in Barker’s Hypothesis and have been referred to as “fetal programming” [4,5]. To this end, correlations have been established between several adipokines levels in cord serum with parameters such as birthweight and gestational age [6,7,8], while little is known about newly emerging adipokines. Transforming growth factor beta-1 (TGFβ1) is one such adipokine, with few studies existing analyzing TGFβ1 levels in maternal and cord serum and their relationship with pregnancy outcomes and fetal growth.

The TGFβ superfamily was first described in 1990 and encompasses several secretory proteins involved in cellular proliferation, differentiation and apoptosis [9]. This large family encompasses TGFβ isoforms, growth differentiation factors, bone morphogenetic proteins, as well as activin/inhibin subfamilies [10]. The first member of this family to be discovered was the adipokine TGFβ1, a 25-kDa, disulfide-linked non-glycosylated homodimer with diverse functions [10]. TGFβ1 has been shown to play a role in endothelial regeneration and wound repair [11,12]. While lower concentrations of TGFβ1 may have an indirect mitogenic effect on epithelial cells, higher concentrations could exert an inhibitory effect on proliferation [13]. Furthermore, TGFβ1 has been found to be released from adipose tissue [14] and is elevated in individuals with obesity [15]. Subsequently, it is also associated with systemic insulin resistance and inflammation [15,16]. Furthermore, TGFβ1 is abundant in the endometrium and seems to be an important factor in the proliferation, apoptosis and regeneration associated with menstruation and decidualization during pregnancy [17]. Review of in vitro data highlighted the cell proliferation- and differentiation-promoting properties of TGFβ1, as well as its resulting associations with embryonic development [18]. Additional sources of TGFβ1 include placenta [17], as well as immune cells such as monocytes and macrophages [19]. TGFβ1 expression by blood cells as well as placenta has also been associated with pregnancy and fertility in humans [20]. More recently, the ability of TGFβ1 to promote a fetal-like regeneration in intestinal epithelium post-irradiation in mouse models was reported [21]. This illustrates the interest in the potential association of TGFβ1 with intrauterine development and growth.

In this study, we aimed to analyze TGFβ1 levels in maternal serum and in cord blood at birth and assess whether TGFβ1 is of maternal or fetal origin. We also investigated the association of maternal and cord serum TGFβ1 with gestational parameters such as gestational weight gain as well as parameters of intrauterine and child growth in the first years of life.

## 2. Materials and Methods

### 2.1. Study Design

This was an observational, longitudinal study on a cohort comprised of 79 healthy mothers and their healthy offspring. We obtained serum samples of pregnant women taken at the 36th week of gestation as well as serum samples from the umbilical cord at birth and measured TGFβ1 levels present in these samples. Furthermore, we assessed the weight progression of offspring at three checkpoint examinations (U1 at birth, U3 at approximately one month of age and U6 at approximately one year of age) and the association of child weight with TGFβ1 levels measured in umbilical serum at birth.

### 2.2. Study Subjects

All subjects were participants in the LIFE Child Study, a population-based longitudinal study in Leipzig, Germany. It was first established in 2011 with the aim of examining the role of genetic, environmental and lifestyle factors on child health, and the first participants were recruited in that same year [22]. Since then, over 4500 children and 1000 pregnant individuals have been recruited [22]. For the purposes of our study, we only included healthy pregnant mothers without underlying health conditions between the ages of 18 and 41, and their healthy offspring. Exclusion criteria for expectant mothers included chronic, chromosomal or syndromal diseases, pregnancy complications such as gestational diabetes and pre-eclampsia, as well as preterm births (<37 weeks gestation). Inclusion criteria for offspring included appropriate for gestational age birthweight (>3rd percentile, weight range in our cohort 2500–4500 g), the absence of syndromal or chromosomal diseases and a 10-min APGAR score ≥ 8.

The respective serum samples included maternal serum samples taken at the 36th week of gestation and cord serum samples taken at birth. In addition, LIFE Child also provided us with both maternal and child biometric measurements and lab values. Maternal parameters included measurements recorded in the pregnancy records of the participating mothers such as weight gain and gestational age at birth. Child parameters comprised of anthropometric measurements and observations recorded in the check-up booklet of children. The check-up booklet encompasses growth and weight development parameters of children measured at standardized assessments (U1-U10) spanning from birth until eight years of age (U1:birth, U2: 72 h after birth, U3: 1 month of age, U4: 3–4 months of age, U5: 6–7 months of age, U6: 1 year of age, U7: 2 years of age, U7a: 3 years of age, U8: four years of age, U9: five years of age, U10: 8 years of age). For our study, we identified 79 mothers recruited during pregnancy and their respective offspring, all of whom met the inclusion criteria and participated in at least three standardized assessments (U1, U3 and U6). Of the 79 children included in our study, we had follow-up measures from 79 participants at the U3 and 78 at U6. Measured values at each of these standardized assessments included weight as well as Kromeyer–Hauschild standard deviation scores (SDS) for weight [23].

### 2.3. Assays and Laboratory Measurements

Blood samples were processed by the Leipzig Medical Biobank team according to standard operating procedures. While samples from pregnant women were processed within about 180 min after blood collection, cord blood samples were prepared for storage during the working hours of the biobank, at the latest in the morning of the next working day. All samples have been stored at −80 °C (2D-barcoded cryotubes (Azenta)) or at temperatures below −150 °C in straws (CryoBiosystems IMV) [24]. Circulating TGFβ1 was quantified in maternal and cord serum using commercial enzyme-linked immunosorbent assays (ELISAs) (R&D Systems©, Minneapolis).

### 2.4. Statistical Analysis

All statistical analyses were performed using GraphPad Prism 9.5.1 and R 4.2.2. Distribution was tested for normality of variables and residuals using the Shapiro–Wilk *W*-test, and variables were logarithmically transformed where deemed necessary. TGFβ1 levels in maternal and cord serum were compared using Mann–Whitney-U Test. The association of both maternal and cord circulating TGFβ1 with various parameters of child growth and maternal gestational parameters was assessed using simple and multiple regression analyses. Regression results were reported as change in the outcome per +10,000 pg/mL TGFβ1. Correlations were assessed with Spearman’s rank correlation analysis. Results were visualized through boxplot, scatterplot and correlation matrix. A *p*-value of <0.05 was considered statistically significant in all analyses.

## 3. Results

Study group characteristics are summarized in Table 1. The mean age of mothers in the study population was 30.0 ± 4.7 years and the mean gestational weight gain during pregnancy was 15.0 ± 5.7 kg. The mean gestational age at birth was 38.8 ± 1.2 weeks. In offspring, mean birthweight was 3445 ± 397.7 g, while mean birth length was 49.9 ± 1.8 cm. At U3, children had a mean age of 0.09 ± 0.03 years (31 days or one month), a mean weight of 4419 ± 579.9 g and a mean length of 54.6 ± 2.0 cm. The mean age at the U6 checkup of children was 0.96 ± 0.07 years. At the U6, mean child weight was 9391 ± 980.7 g and mean length was 74.7 ± 2.7 cm.

### 3.1. Association between Maternal and Cord TGFβ1 Levels

**Figure 1:**
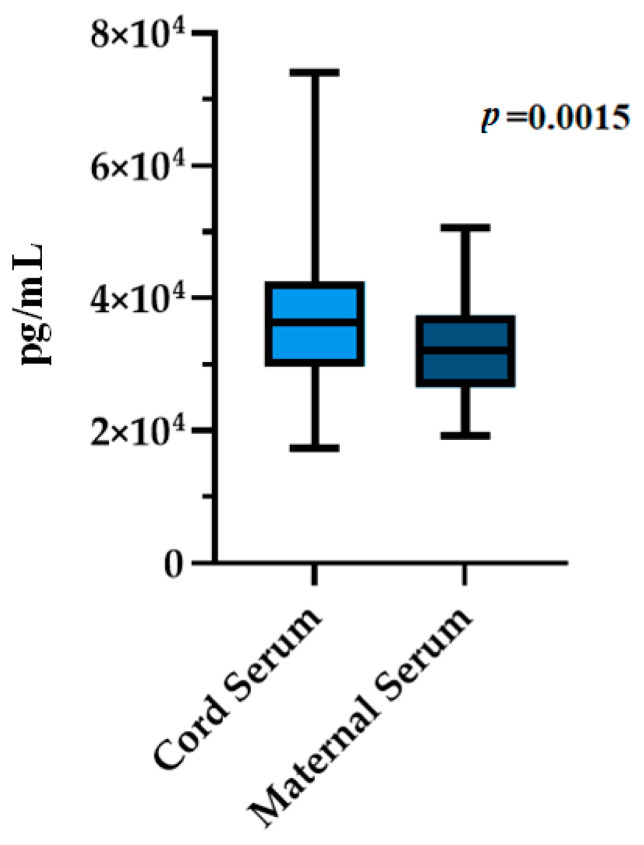
TGFβ1 concentrations in maternal and cord serum, n = 79.

The mean maternal serum TGFβ1 concentration at 36 weeks gestation was 32,310 ± 7073 pg/mL, while mean TGFβ1 concentration in cord blood at birth was 37,738 ± 11,186 pg/mL. A Mann–Whitney *U*-test showed that TGFβ1 levels in cord blood were significantly higher than in maternal serum at 36 weeks gestation (W = 2211, *p* = 0.002) (Figure 1).

### 3.2. Association between Maternal TGFβ1 Levels and Child Growth

No significant correlations were found between maternal TGFβ1 levels at 36 weeks gestation and child weight or BMI at birth, at roughly one month of age, nor at approximately one year of age.

### 3.3. Association between Cord TGFβ1 Levels and Child Growth

**Figure 2:**
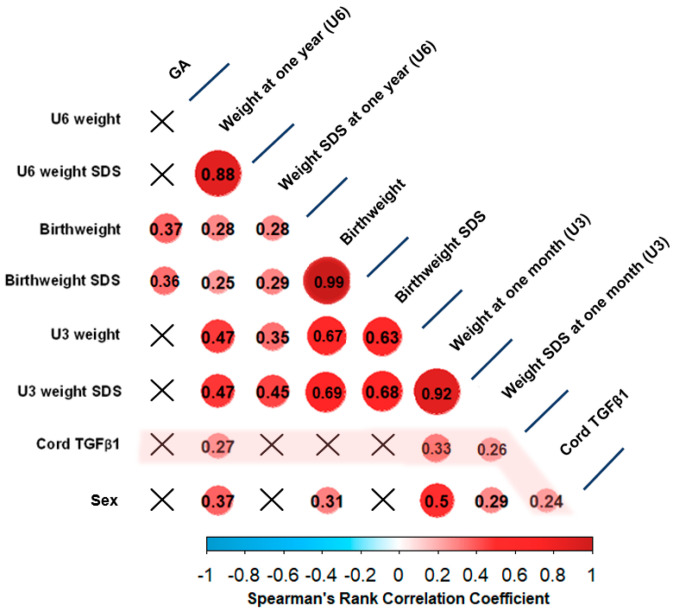
**Spearman’s rank correlation matrix of cord TGFβ1 levels and child growth parameters**: Crossed-out areas symbolize insignificant correlations, while circles symbolize significant correlations. The color of each circle corresponds to Spearman’s rank correlation coefficient (see legend at bottom of figure), which is further provided in numerical form. The correlations of cord TGFβ1 are highlighted in pink. Significant positive correlations were found between cord TGFβ1 levels at birth and child weight at U3, child weight SDS at U3, as well as child weight at U6. Circles represent significant correlations while blank areas represent insignificant correlations. The color of each circle represents the respective Spearman’s rank correlation coefficient (see legend).

Spearman’s rank correlation test did not reveal any significant correlations between cord TGFβ and child weight parameters at birth. Furthermore, no significant correlations could be found between cord TGFβ1 and gestational age at birth. However, TGFβ1 in cord serum at birth correlated significantly with biological sex (r = 0.239, *p* = 0.033). Furthermore, we found statistically significant positive correlations between cord TGFβ1 levels at birth and child weight as well as weight SDS at U3 (1 month) (r = 0.335, *p* = 0.003 and r = 0.235, *p* = 0.04, respectively). In line with this finding, child weight at U6 (1 year) also correlated positively and significantly with cord TGFβ1 (r = 0.273, *p* = 0.016), although correlation of cord TGFβ1 and weight SDS at this age was not significant. Child sex also correlated significantly and positively with child weight at birth (r = 0.308, *p* = 0.006), at U3 (r = 0.498, *p* < 10^−4^) and at U6 (r = 0.373, *p* = 0.001). All correlations are presented in Figure 2.

**Figure 3:**
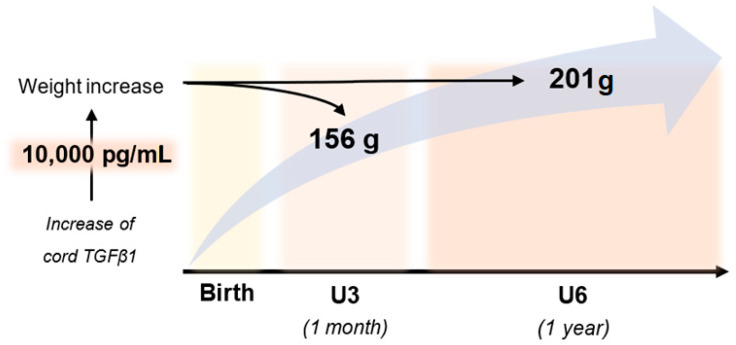
Child weight increase adjusted for sex associated with an increase of 10,000 pg/mL in cord TGFβ1 at birth: 156 g at one month of age and 201 g at one year of age.

**Figure 4:**
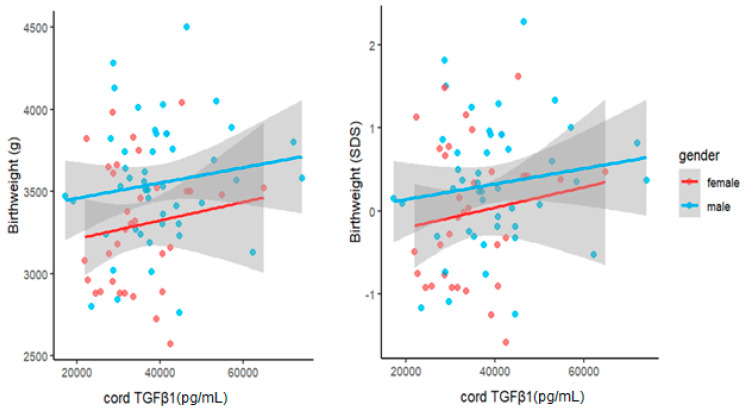
Scatter plot of raw child birthweight (**left**) as well as birthweight SDS (**right**) with cord TGFβ1 levels at birth, considering both females (red) and males (blue). Males appear to have a higher raw birth weight as well as birthweight SDS Score when compared to females.

**Figure 5:**
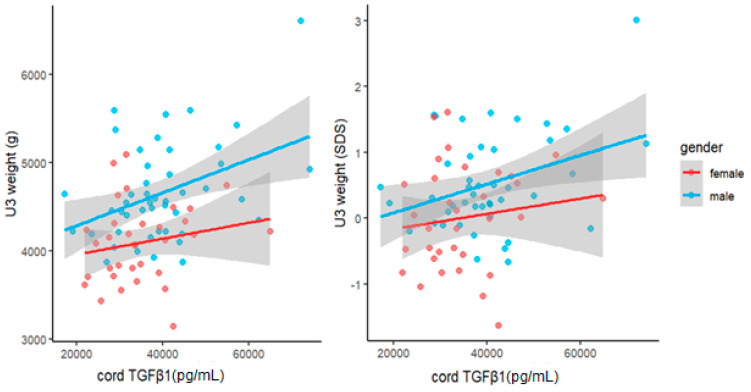
Scatter plot of raw child weight at U3 (1 month) (**left**) as well as weight SDS (**right**) with cord TGFβ1 levels at birth, considering both females (red) and males (blue). Cord TGFβ1 levels seem to increase more drastically with increasing raw birth weight in males when compared to those of females.

**Figure 6:**
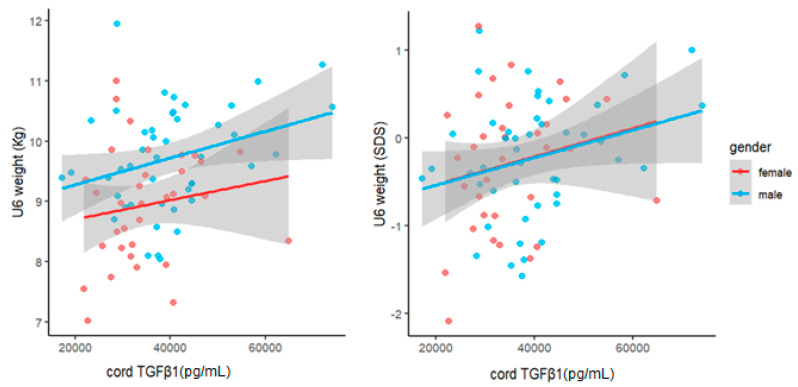
Scatter plot of raw child weight at U6 (1 year) (**left**) as well as weight SDS (**right**) with cord TGFβ1 levels at birth, considering both females (red) and males (blue). Here, cord TGFβ1 levels associated with increasing weight SDS appear to be similar in both males and females.

To investigate the relationship between cord levels of TGFβ1 at birth and child weight development during the first year of life, we performed univariate and multivariate linear regression analyses. Cord TGFβ1 was positively related to the child’s weight at birth, U3 (1 month) and U6 (1 year). However, the significance of associations was not consistent across the different measures. At birth, birthweight and the related SDS were only significantly related to TGFβ1 when correcting for GA (β_10,000 TGFβ1_ = 88, *p* = 0.03 and β < 10^−4^, *p* < 10^−4^ respectively). Further, birthweight was also significantly related to cord TGFβ1 when adjusted for child sex (β_10,000 TGFβ1_ = 50, *p* = 0.02). At U3 and U6, both measures showed significant associations with TGFβ1, that persisted even after adjustment for sex and, in the case of the raw weight measure, birthweight. At U3, we found the following effect sizes after adjusting for sex: weight: β_10,000 TGFβ1_ = 156, *p* = 0.003 and weight SDS: β < 10^−4^, *p* = 0.02. At U6, we found similar associations between cord TGFβ1 and weight (β_10,000 TGFβ1_ = 201 g, *p* = 0.032) as well as weight SDS (β < 10^−4^, *p* = 0.033). Accordingly, an increase of 10,000 pg/mL in cord TGFβ1 concentrations at birth was associated with a weight increase of 201 g at roughly one year of age when adjusted for child sex (Figure 3). In order to further visualize our data, we also performed scatter plots of child weight and cord TGFβ1 levels at birth (Figure 4, Figure 5 and Figure 6). The results of multivariate regression analyses between cord TGFβ1 and child birthweight adjusted for gestational age as well as child weight adjusted for sex are summarized in Table 2. The results of all multivariate as well as univariate regression analyses are provided in the Supplementary Materials (Tables S1–S6). The values of all correlations performed on cord TGFβ1 levels at birth and child weight parameters are summarized in Table S7 in the Supplementary Materials.

### 3.4. Association between Maternal and Cord TGFβ1 Levels and Maternal Parameters

No correlations were found between maternal serum TGFβ1 levels at 36 weeks gestation and maternal gestational parameters including pre-gestational weight, maximal gestational weight gain and maternal age at birth. Similarly, no significant associations were found between cord TGFβ1 levels at birth and the aforementioned maternal parameters.

## 4. Discussion

To better understand the role of TGFβ1 in human fetal growth, we attempted to determine the source of cord TGFβ1. The higher levels of TGFβ1 present in cord blood at birth in comparison to TGFβ1 levels found in maternal serum at 36 weeks gestation suggest that the source of cord TGFβ1 is more likely fetal and point to its possible role in intrauterine growth. To our knowledge, this is the first study comparing paired maternal and cord TGFβ1 levels at birth, however similar studies have been carried out on adipokines other than TGFβ1. For example, Briana et al. reported higher levels of umbilical cord preadipocyte factor-1 (pref-1) at partum in comparison to levels found in maternal serum antepartum, indicating the fetal origin of cord pref-1 [25]. Likewise, lipocalin-2 was also found to be increased in cord serum and is therefore most likely of fetal origin [26]. As our cohort included exclusively healthy, term babies born at an appropriate weight (2500–4500 g) for gestational age (AGA), we speculate that fetal secretion of TGFβ1 is a physiological process and is associated with normal fetal and child development.

Higher levels of cord TGFβ1 in comparison to maternal serum have also been reported in recent studies. In a study assessing TGFβ1 levels in maternal serum in the second and third trimesters compared to non-pregnant controls, Power et al. reported elevated maternal TGFβ1 at both trimesters when compared with the non-pregnant state. They found TGFβ1 to reach its highest level in late gestation. One major difference compared to our study, was the use of unrelated mothers and children instead of the respective offspring of the included mothers. Nonetheless, in accordance with our findings, they also reported higher levels of TGFβ1 in cord serum when compared to levels found in maternal serum [27]. Furthermore, cord TGFβ1 was significantly higher in the time before labor than after labor, pointing to a regulatory function in the maternal immune response against the fetus. In contrast, higher levels of cord blood TGFβ1 were reported in healthy offspring born after spontaneous vaginal delivery at term when compared to healthy offspring delivered via elective cesarean section in two studies [28,29]. Here, the authors ventured that a physiological cytokine release evoked by vaginal delivery may also result in elevated cord TGFβ1 levels at birth.

The origin of fetal TGFβ1 has also been investigated. High expression of TGFβ1 has been reported in the syncytiotrophoblast cells of the placental villi [17,30], and it was speculated that TGFβ1 potentially hinders cytotrophoblast fusion and syncytialisation in physiological gestation [31].

In our findings, maternal serum TGFβ1 did not correlate with the pregestational weight nor with the gestational weight gain of mothers. However, TGFβ1 has been associated with obesity and weight gain in adults. Significant associations have been found between TGFβ1 levels and hepatic abnormalities such as steatosis, obesity, and CRP [15]. In addition, associations between TGFβ1 and insulin resistance and the development, differentiation and function of pancreatic islet β-cells have also been found [16,32]. Similarly, TGFβ1 secretion was found to be greater in the adipose tissue of individuals with obesity when compared to a control group of individuals with normal BMI [14]. TGFβ1 methylation was decreased in the saliva of pregnant women who were obese in comparison to non-pregnant controls with normal BMI, suggesting an upregulation of TGFβ1 associated with obesity and gestation [33]. These data do not contradict our results, because our cohort only included mothers with a normal BMI. We postulate that associations of TGFβ1 with adipose tissue and its higher circulating concentrations in individuals with obesity may only apply to states of active inflammation and obesity, and not to individuals with normal BMI.

Our study was the first of its kind to investigate the relationship between cord TGFβ1 at birth and child growth during the first year of life. The longitudinal study design allowed us to assess long term outcomes in addition to immediate outcomes at birth. We found significant associations between cord TGFβ1 at birth and birthweight, as well as birthweight SDS after adjusting for male gender and gestational age.

Recent studies have also explored the relationship between cord TGFβ1 and child parameters at birth, albeit not in a longitudinal fashion. In a previous study, infants with intrauterine growth restriction (IUGR) displayed elevated cord TGFβ1 at birth in comparison to a matched AGA group [29]. This increase in cord TGFβ1 was proposed to result from the abnormal fetal perfusion associated IUGR pregnancies. Moreover, the TGFβ1 gene was identified as a possible contributor gene in placental insufficiency and IUGR [34]. Placental samples assessed from small for gestational age (SGA) pregnancies (defined as birth weight of less than the 10th percentile for gestational age) and normal gestations reported similar placental TGFβ1 gene expression in both groups [35]. In a further study, placental TGFβ1 gene expression SGA pregnancies was reported to be similar to that of normal pregnancies at birth [35]. This suggests an upregulation of cord TGFβ1 in the case of pathophysiological pregnancy states such as IUGR, but not in normal variants of intrauterine growth such as SGA. Our findings, on the other hand, further show a significant positive correlation between cord TGFβ1 at birth and growth parameters at one month and one year of age. Taken together, our findings suggest that cord TGFβ1 at birth is associated not only with child weight at birth, but also with long-term fetal outcome in the first year of age. This could implicate TGFβ1 as a possible player in the fetal programming of fetal metabolic processes, with its levels perhaps being predetermined based on the intrauterine environment and thus influencing child weight gain in the first year of life.

Intrauterine conditions have been shown to shape the health trajectory of offspring through fetal programming [36,37,38]. While we did not find any studies evaluating TGFβ1 with child growth after birth, several studies have been published considering the role of other adipokines in child growth. For example, cord leptin at birth was found to correlate negatively with infant weight gain from birth to four months of age, and this negative correlation was evident even at two years of age [39].

In our study, no significant associations between maternal TGFβ1 levels and offspring birth parameters, such as weight, length, head circumference or BMI could be described. Nevertheless, several studies investigated maternal TGFβ1 levels and gestational outcomes with some significant findings. Negative associations were found between maternal TGFβ1 concentrations and birthweight as well as head circumference of offspring [40]. Moreover, decreased maternal serum TGFβ1 concentrations have also been observed in SGA gestations in the 34th gestational week, however this decrease disappeared at the 38th week [41]. Similarly, a study evaluating maternal TGFβ1 levels in pregnant women with pre-eclampsia and healthy pregnant controls did not find a significant difference [42]. Taken together, it appears that cord TGFβ1 is the better candidate for assessing intrauterine and child growth in comparison to maternal TGFβ1.

Interestingly, while cord TGFβ1 levels at birth were significantly associated with child sex in our healthy cohort, a study investigating TGFβ1 gene expression in the placentas of SGA pregnancies found no differences in gene expression between males and females [35]. Although the higher TGFβ1 levels in males yielded in our analyses may simply be due to the larger weight of males, cord TGFβ1 levels at birth seem to be associated with child weight at one year of age independent of gender.

### Study Limitations

While our cohort was relatively large with 79 mother–child pairs, this number still restricts the generalizability and creates a higher risk of type II errors. Moreover, we did not perform a sample size calculation, as it is generally difficult to attain a large cohort of pregnant mothers and their offspring without underlying health conditions or complications during pregnancy and at birth as well as follow-up examinations up to a year after birth. Although our cohort was largely homogenous in terms of birth weight and gestational age, further factors that may impact a healthy pregnancy such as maternal diet, environmental factors, or exposure to allergens during pregnancy were not considered. Of over 4500 children recruited by LIFE Child since 2011, we were only able to identify 79 children who met our inclusion criteria. Additionally, although we compared maternal and cord serum samples, there was a gap between maternal serum sampling at 36 weeks gestation and cord serum sampling at birth. This approximate window of four weeks allows a margin for fluctuations in TGFβ1 levels. The slight variance in age between children at the respective examinations renders anthropometric measurements liable to error. Furthermore, the cross-sectional nature of the serum samples we acquired meant that we were unable to assess TGFβ1 longitudinally. Changes in TGFβ1 in maternal serum during pregnancy could provide more explanation on the associations of TGFβ1 with parameters of intrauterine growth. Similarly, measurement of pre-pregnancy and antepartum TGFβ1 levels in maternal serum could have also been useful in investigating the role of this adipokine in metabolic processes during gestation.

The findings of our study raise several questions that could be tackled in the future. Future research directions include a longitudinal assessment of maternal serum TGFβ1 levels throughout pregnancy, as well as TGFβ1 levels in the breastmilk of mothers, especially in cases where they choose to breastfeed. Similarly, longitudinal assessment of child serum TGFβ1 levels throughout childhood would also be beneficial to study the association of TGFβ1 with child weight development, however blood samples are rarely drawn from healthy children without therapeutic consequence. Further, our study only considered healthy pregnancies and births; it would be of interest to observe whether cord TGFβ1 levels are also associated with child weight development in SGA or LGA pregnancies, or whether pregnancy diseases such as gestational diabetes or pre-eclampsia affect this association in any way.

## 5. Conclusions

In summary, our study suggests that TGFβ1 detected in cord serum at birth is most likely of fetal origin. Furthermore, we found a significant relationship between cord TGFβ1 levels at birth and child weight development in the first year of life. These associations were more pronounced at one month of age than at birth. Child weight at approximately one year of age correlated significantly with cord TGFβ1 levels at birth. Accordingly, an increase of 10,000 pg/mL in cord TGFβ1 concentration at birth was associated with an average weight increase of 156 g at one month of age and 201 g at one year of age when adjusted for gender. Further research considering child TGFβ1 concentrations and child growth parameters is needed to elucidate the role of TGFβ1 in growth and weight gain.

## Figures and Tables

**Table 1:** Study group parameters.

		Mean	Min	Max	25th Quartile	75th Quartile	SD
Maternal Parameters: n = 79							
Maternal age	* years*	30.02	18.74	41.55	26.69	32.45	4.649
GWG (n = 72)	* kg*	14.97	3.000	31.00	11.00	17.50	5.729
GA	* weeks*	39.80	37.00	41.70	38.70	40.90	1.218
Serum TGFβ1 at 36 Weeks gestation	* pg/mL*	32,310	19,172	50,542	26,480	37,453	7073
Child Parameters at birth: n = 79: female = 33, male = 46							
Weight	* g*	3445	2570	4500	3160	3740	397.7
Length	* cm*	49.86	46.00	55.00	49.00	51.00	1.824
BMI	* kg/m^2^*	13.83	11.20	16.71	13.05	14.56	1.148
Cord TGFβ1	* pg/mL*	37,738	17,238	74,115	29,696	42,534	11,186
Child Parameters at U3: n = 79							
Age	* years*	0.086	0.024	0.153	0.064	0.109	0.030
Weight	* g*	4419	3140	6600	4070	4700	579.9
Length	* cm*	54.61	49.40	59.50	53.00	56.00	2.008
BMI	* kg/m^2^*	14.77	12.20	19.76	13.79	15.57	1.340
Child Parameters at U6: n = 78							
Age	* years*	0.959	0.766	1.177	0.9075	0.999	0.074
Weight	* g*	9391	7020	11,940	8699	10,113	980.7
Length	* cm*	74.73	68.00	80.00	73.00	76.50	2.708
BMI	* kg/m^2^*	16.79	14.15	20.09	15.82	17.63	1.255

GWG = gestational weight gain, GA = gestational age, SD = standard deviation.

**Table 2:** Multivariate regression analysis of child weight parameters with cord TGFβ1 adjusted for gestational age.

		β_10,000_	*p*	R^2^ Adjusted
**Variable:** Birthweight				0.20
**Predictor:**	Cord TGFβ1	88	0.02	
	GA	14 × 10^5^	<10^−3^	
**Variable:** U3 weight (1 month)				0.30
**Predictor:**	Cord TGFβ1	156	<10^−2^	
	Male sex	49 × 10^5^	<10^−3^	
**Variable:** U6 weight (1 year)				0.19
**Predictor:**	Cord TGFβ1	201	0.03	
	Male sex	68 × 10^5^	<10^−2^	

## Data Availability

The dataset presented in this article cannot be shared publicly because of ethical restrictions. The LIFE Child study is a study collecting potentially sensitive information. Publishing data is not covered by the informed consent provided by the study participants. Furthermore, the data protection concept of LIFE requires all (external as well as internal) researchers interested in accessing data to sign a project agreement. Researchers interested in accessing data from the LIFE Child study may contact the study by writing to forschungsdaten@medizin.uni-leipzig.de.

## Supplementary Materials

The following supporting information can be downloaded at: https://www.mdpi.com/article/10.3390/biomedicines11082220/s1. Table S1: Multivariate regression analysis of birthweight with cord TGFβ1. Table S2: Univariate regression analysis of birthweight with cord TGFβ1. Table S3: Multivariate regression analysis of weight at 1 month (U3) with cord TGFβ1. Table S4: Univariate regression analysis of weight at 1 month (U3) with cord TGFβ1. Table S5: Multivariate regression analysis of weight at 1 year (U6) with cord TGFβ1. Table S6: Univariate regression analysis of weight at 1 year (U6) with cord TGFβ1. Table S7: Spearman’s Rank Correlation of cord TGFβ1 levels and child growth parameters.
