# Supplementary material for: Association between TGFβ1 Levels in Cord Blood and Weight Progress in the First Year of Life
Source: Biomedicines. 2023 Aug 8;11(8):2220. doi: 10.3390/biomedicines11082220 (PMC10452394; doi:10.3390/biomedicines11082220)
Supplement: Supplementary file 1 [file biomedicines-11-02220-s001.zip › biomedicines-2533385-supplementary.pdf]

Table S1: Multivariate regression analysis of birthweight with cord TGFβ1

		$\beta_{10,000}$	p	R^2 adjusted
**Variable:** Birthweight				0.10
**Predictors:**	Cord TGFβ1	50	0.21	
	Male sex	23e+5	0.01*	
**Variable:** Birthweight				0.20
**Predictors:**	Cord TGFβ1	88	0.02*	
	GA	14e+5	$<10^{-3}$*	
**Variable:** Birthweight SDS				0.04
**Predictors:**	Cord TGFβ1	$<10^{-3}$	0.22	
	Male Sex	2940	0.11	
**Variable:** Birthweight SDS				0.19
**Predictors:**	Cord TGFβ1	$<10^{-3}$	0.03*	
	GA	2784	$<10^{-3}$*	

Table S2: Univariate regression analysis of birthweight with cord TGFβ1

		$\beta_{10,000}$	p	R^2 adjusted
**Variable:** Birthweight				0.02
**Predictor:**	Cord TGFβ1	72	0.07	
**Variable:** Birthweight SDS				0.02
**Predictor:**	Cord TGFβ1	$<10^{-3}$	0.11	

Table S3: Multivariate regression analysis of weight at 1 month (U3) with cord TGFβ1

		$\beta_{10,000}$	p	R^2 adjusted
**Variable:** U3 weight				0.30
**Predictors:**	Cord TGFβ1	156	$<10^{-2}$*	
	Male sex	49e+5	$<10^{-3}$*	
**Variable:** U3 weight				0.53
**Predictors:**	Cord TGFβ1	136	$<10^{-2}$*	
	Birthweight	9325	$<10^{-4}$*	
**Variable:** U3 weight SDS				0.15
**Predictors:**	Cord TGFβ1	$<10^{-3}$	0.02*	
	Male Sex	4189	0.02*	

Table S4: Univariate regression analysis of weight at 1 month (U3) with cord TGFβ1

		$\beta_{10,000}$	p	R^2 adjusted
**Variable:** U3 weight				0.14
**Predictor:**	Cord TGFβ1	203	$<10^{-3}$*	
**Variable**: U3 weight SDS				0.09
**Predictor:**	Cord TGFβ1	$<10^{-3}$	$<10^{-2}$	

SDS: Standard Deviation Score (Kromeyer-Hauschild), GA: gestational age, U3=Examination at 1 month of age, U6: Examination at 1 year of age

Table S5: Multivariate regression analysis of weight at 1 year (U6) with cord TGFβ1

		$\beta_{10,000}$	**p**	**R^2 adjusted**
**Variable:** U6 weight				0.19
**Predictors:**	Cord TGFβ1	201	0.03*	
	Male sex	68e+5	$<10^{-2}$*	
**Variable:** U6 weight				0.12
**Predictors:**	Cord TGFβ1	227	0.02*	
	Birthweight	5669	0.04*	
**Variable:** U6 weight SDS				0.04
**Predictors:**	Cord TGFβ1	$<10^{-3}$	0.03*	
	Male Sex	-138	0.93	

Table S6: Univariate regression analysis of weight at 1 year (U6) with cord TGFβ1

		$\beta_{10,000}$	**p**	**R^2 adjusted**
**Variable:** U6 weight				0.08
**Predictor:**	Cord TGFβ1	268	$<10^{-2}$*	
**Variable**: U6 weight SDS				0.05
**Predictor:**	Cord TGFβ1	$<10^{-3}$	0.03*	

SDS: Standard Deviation Score (Kromeyer-Hauschild), GA: gestational age, U3=Examination at 1 month of age, U6: Examination at 1 year of age

Table S7: Spearman's Rank Correlation of cord TGFβ1 levels and child growth parameters

	GA	U6 Weight	U6 Weight SDS	Birthweight	Birthweight SDS	U3 Weight	U3 Weight SDS	Cord TGFβ1	Male Sex
		**P Values**							
**GA**		0.089	0.067	**0.001**	**0.001**	0.054	0.078	0.460	0.433
**U6 Weight**	-		**<0.001**	**0.012**	**0.028**	**0.000**	**0.000**	**0.016**	**0.001**
**U6 Weight SDS**	-	0.884		**0.012**	**0.010**	**0.002**	**0.000**	0.074	0.791
**Birthweight**	0.370	0.285	0.283		**<0.001**	**<0.001**	**<0.001**	0.076	**0.006**
**Birthweight SDS**	0.362	0.249	0.292	0.989		**<0.001**	**<0.001**	0.102	0.082
**U3 Weight**	-	0.470	0.347	0.671	0.627		**<0.001**	**0.003**	**<0.001**
**U3 Weight SDS**	-	0.474	0.448	0.694	0.683	0.922		**0.040**	**0.011**
**Cord TGFβ1**	-	0.273	-	-	-	0.335	0.235		**0.034**
**Male Sex**	-	0.373	-	0.308	-	0.498	0.287	0.239	
	**Spearman's Rank Correlation Coefficient R**								

P values are shown in blue, Spearman's Rank Correlation coefficient is shown in red for significant correlations
SDS: Kromeyer-Hauschild Standard Deviation Score, GA: gestational age, U3: Examination at 1 month, U6: Examination at 1 year
